# Mindfulness starts with the body: somatosensory attention and top-down modulation of cortical alpha rhythms in mindfulness meditation

**DOI:** 10.3389/fnhum.2013.00012

**Published:** 2013-02-13

**Authors:** Catherine E. Kerr, Matthew D. Sacchet, Sara W. Lazar, Christopher I. Moore, Stephanie R. Jones

**Affiliations:** ^1^Department of Family Medicine, Brown UniversityProvidence, RI, USA; ^2^Neurosciences Program, Stanford University School of MedicineStanford, CA, USA; ^3^Department of Psychology, Stanford UniversityStanford, CA, USA; ^4^Athinoula A. Martinos Center For Biomedical Imaging, Mass General HospitalCharlestown, MA, USA; ^5^Department of Neuroscience, Brown UniversityProvidence, RI, USA

**Keywords:** alpha rhythm, attention, chronic pain, depression relapse, mindfulness meditation, somatosensory cortex, thalamocortical loop

## Abstract

Using a common set of mindfulness exercises, mindfulness based stress reduction (MBSR) and mindfulness based cognitive therapy (MBCT) have been shown to reduce distress in chronic pain and decrease risk of depression relapse. These standardized mindfulness (ST-Mindfulness) practices predominantly require attending to breath and body sensations. Here, we offer a novel view of ST-Mindfulness's somatic focus as a form of training for optimizing attentional modulation of 7–14 Hz alpha rhythms that play a key role in filtering inputs to primary sensory neocortex and organizing the flow of sensory information in the brain. In support of the framework, we describe our previous finding that ST-Mindfulness enhanced attentional regulation of alpha in primary somatosensory cortex (SI). The framework allows us to make several predictions. In chronic pain, we predict somatic attention in ST-Mindfulness “de-biases” alpha in SI, freeing up pain-focused attentional resources. In depression relapse, we predict ST-Mindfulness's somatic attention competes with internally focused rumination, as internally focused cognitive processes (including working memory) rely on alpha filtering of sensory input. Our computational model predicts ST-Mindfulness enhances top-down modulation of alpha by facilitating precise alterations in timing and efficacy of SI thalamocortical inputs. We conclude by considering how the framework aligns with Buddhist teachings that mindfulness starts with “mindfulness of the body.” Translating this theory into neurophysiology, we hypothesize that with its somatic focus, mindfulness' top-down alpha rhythm modulation in SI enhances gain control which, in turn, sensitizes practitioners to better detect and regulate when the mind wanders from its somatic focus. This enhanced regulation of somatic mind-wandering may be an important early stage of mindfulness training that leads to enhanced cognitive regulation and metacognition.

## Introduction

As a form of mental training, mindfulness meditation has been practiced for over two millennia. Originating in Asian Buddhist traditions, the practice is said to involve the cultivation of experiential awareness of the present moment (Brown, 2003; Analayo, 2004). This present-moment focus is thought to improve well-being by allowing individuals to become aware of sensations, emotions and thoughts that arise in the mind without judgment or reactivity (Baer, 2003; Bishop, 2004). Over the last two decades, mindfulness-related treatments have become an increasingly common component of the healthcare system in developed countries through therapies such as dialectical behavior therapy (Linehan, 1993), acceptance and behavior therapy (Hayes et al., 1999), mindfulness based cognitive therapy (MBCT) (Teasdale et al., 2000a,b) and mindfulness based stress reduction (MBSR) (Kabat-Zinn, 1990).

MBCT and MBSR use a standardized form of mindfulness meditation practice (ST-Mindfulness) that has been extensively tested in randomized controlled trials (Fjorback et al., 2011). The formal practice-based content of MBSR and MBCT are nearly identical: The programs share an 8-week instructional format that involves three somatically focused meditative techniques (body scan, sitting meditation, and mindful yoga) that are thought to help participants cultivate non-judgmental, mindful awareness of present-moment experience.

Based on multiple randomized clinical trials, there is good evidence for the efficacy of these ST-Mindfulness programs for preventing mood disorders in people at high risk of depression (Teasdale et al., 2000a,b; Ma and Teasdale, 2004; Segal et al., 2010; Fjorback et al., 2011; Piet and Hougaard, 2011), improving mood and quality of life in chronic pain conditions such as fibromyalgia (Grossman et al., 2007; Sephton et al., 2007; Schmidt et al., 2011) and low-back pain (Morone et al., 2008a,b), in chronic functional disorders such as IBS (Gaylord et al., 2011) and in challenging medical illnesses, including multiple sclerosis (Grossman et al., 2010) and cancer (Speca et al., 2000). ST-Mindfulness has also been shown to decrease stress in healthy people undergoing difficult life situations (Cohen-Katz et al., 2005), such as caring for a loved-one with Alzheimer's disease (Epstein-Lubow et al., 2006).

As previous reviewers have noted (Holzel et al., 2011; Slagter et al., 2011; Vago and Silbersweig, 2012), therapeutic benefits of ST-Mindfulness training extend across a broad range of conditions. Numerous behavioral and neural mechanisms have been proposed to explain these positive outcomes. Proposed mechanisms include changes in neural networks underlying emotion regulation (Holzel et al., 2008), illustrated by findings showing decreased amygdala response after ST-Mindfulness in social anxiety patients exposed to socially threatening stimuli (Goldin and Gross, 2010). Other neural mechanisms highlighted in recent reviews include changes in self-processing (Vago and Silbersweig, 2012) based on multiple studies including a report showing decreases in activation in midline cortical areas used in self-related processing in ST-Mindfulness trained subjects (Farb et al., 2007). Given these extant comprehensive reviews, our goal here is a rather more simple and pragmatic effort to answer the question: how does the specific ST-Mindfulness training sequence in somatically focused attention in body and breath focused meditative exercises lead to such a broad range of benefits?

A clue as to how ST-Mindfulness affects mood and distress comes from findings that it leads to beneficial changes in cognitive processing in people with mood disorders, chronic functional disorders and chronic pain. Thus, ST-Mindfulness is reported to reduce self-reported rumination (Ramel, 2004; Deyo et al., 2009), which is the negative repetitive, self-related internal cognitions that predominate in major depression (Nolen-Hoeksema, 2000). In chronic pain and functional disorders, ST-Mindfulness is reported to reduce patients' tendency to catastrophize and engage in repetitive negative cognitions such as, the pain is “terrible and I feel it's never going to get better” (Garland et al., 2012).

Based on these self-reports of decreased rumination and related findings, numerous reviews (Bishop, 2002; Shapiro et al., 2006; Willettt, 2011) have converged on *metacognition* (Teasdale et al., 2002) [insight into one's own thinking process, sometimes described as “decentering” (Roemer and Orsillo, 2003) or “reperceiving” (Shapiro et al., 2006)] as a grand-mechanism underlying ST-Mindfulness efficacy. According to this view, metacognition is an emergent property of mindfulness practice in ST-Mindfulness that is derived from training in subsidiary mechanistic processes including attention and emotion regulation. Drawing on this emergent metacognitive capacity, ST-Mindfulness practitioners learn to monitor their moment-by-moment experience so that they can “step back” from negative, distressing thoughts and feelings in order to view them as “mental events” rather than as unmediated reflections of reality.

But how does metacognitive insight arise from the specific practices trained in ST-Mindfulness? To answer this question, some have suggested (as, for example, in Bishop, 2002), that metacognition in ST-Mindfulness is acquired by enhancing pre-existing modules dedicated to monitoring and controlling cognition. However, this and other similar models of metacognition and mindfulness do not relate the emergence of metacognition to the specific practices trained in ST-Mindfulness.

## ST-mindfulness 8-week practice sequence

Here we lay out a neural framework to explain how ST-Mindfulness training in body-focused attention could exert “upward” influence on metacognition and on cognitive and emotion regulation.

First, it is important to take note of the extent to which the 8-week ST-Mindfulness practice sequence is focused on somatic sensations as described in (Williams et al., 2006) authors of numerous benchmark ST-Mindfulness clinical trials (Teasdale et al., 2000a,b, 2002; see also, Philippot et al., 2012). In the first 2 weeks of the 8-week ST-Mindfulness sequence, all formal practice is devoted to a meditative body scan practice of “moving a focused spotlight of attention from one part of the body to another.” Through this exercise, practitioners are said to learn to feel (1) how to control the attentional spotlight even when focusing on painful, aversive sensations (2) how even *familiar body sensations change and fluctuate* from moment to moment.

In the last 5–6 weeks of class, participants continue to use embodied practices, especially sitting meditation focused on sensations of breathing. These embodied practices are said to teach practitioners (1) how to directly *feel* when the mind has wandered from its sensory focus (2) how to use an intimate familiarity with the fluctuations of sensations of breathing (such as the up and down flow of the breath) as a template for regarding the arising and passing of distressing, aversive thoughts as “mental events” rather than as “facts or central parts of their identity.”

The sequence described by Williams et al. (2006) leads us to propose that these concrete, somatically focused practices of ST-Mindfulness offer training in controlling the attentional spotlight, using subtle tactile and interoceptive feedback to detect when the mind has wandered from its sensory focus and attuning to subtle fluctuations in what had been viewed as unchanging sensory experience. Over time, during the 8-week ST-Mindfulness sequence, these skills learned via this somatic attentional practice become generalized across all of the sensory modalities and also are applied to thoughts, such that practitioners learn to recognize and work with thoughts as “mental events” that arise and pass in the mind. Taken together, these skills provide a sensory-attentional foundation for the cultivation of metacognition.

At the neural level, according to this framework, the somatic focus in ST-Mindfulness elicits changes in brain dynamics that enhance signal-to-noise ratio in sensory-attentional processing. Specifically, we propose that body-focused attentional practice in ST-Mindfulness enhances localized attentional control over the 7–14 Hz alpha rhythm that is thought to play a key role in regulating sensory input to sensory neocortex and in enhancing signal-to-noise properties across the neocortex. Beginning with the enhanced modulation of localized alpha rhythms trained in localized somatic attention practices such as the body-scan, and then proceeding through the 8-week sequence to learn broader modulation of entire sensory modalities (e.g., “whole body attention”) practitioners train in filtering and prioritizing the flow of information through the brain.

On a neural level, ST-Mindfulness training in a highly extendable mechanism of alpha modulation may account for how ST-Mindfulness, which is centered on a *specific* set of low-level sensory-attentional meditative tasks, achieves such a *general* range of positive therapeutic outcomes, possibly by engaging prefrontal cortical areas known to be crucial regulators of thalamocortical circuits during attentionally demanding tasks. This view of localized SI alpha modulation training as an enhancer of prefrontal attentional control is consistent with studies showing long-term changes in ST-Mindfulness practitioners in prefrontal cortex (Davidson et al., 2003; Farb et al., 2007, 2010).

The scientific framework outlined here describes ST-Mindfulness's putative role in enhancing top-down regulation of a 7–14 Hz cortical oscillation, the alpha rhythm that is inversely correlated with spatial attention and is thought to filter the processing of *irrelevant sensory inputs* in primary sensory cortex (Foxe and Snyder, 2011). The attentional focus on body sensations in SI may provide an intuitively available system for learning how to use attention to modulate the alpha rhythm in a manner that bootstraps to other thalamocortical circuits. The generalization of attentional alpha rhythm modulation to other thalamocortical circuits is a possible mechanism by which ST-Mindfulness may enhance the ability to filter and prioritize the flow of information throughout the brain.

**Figure 1:**
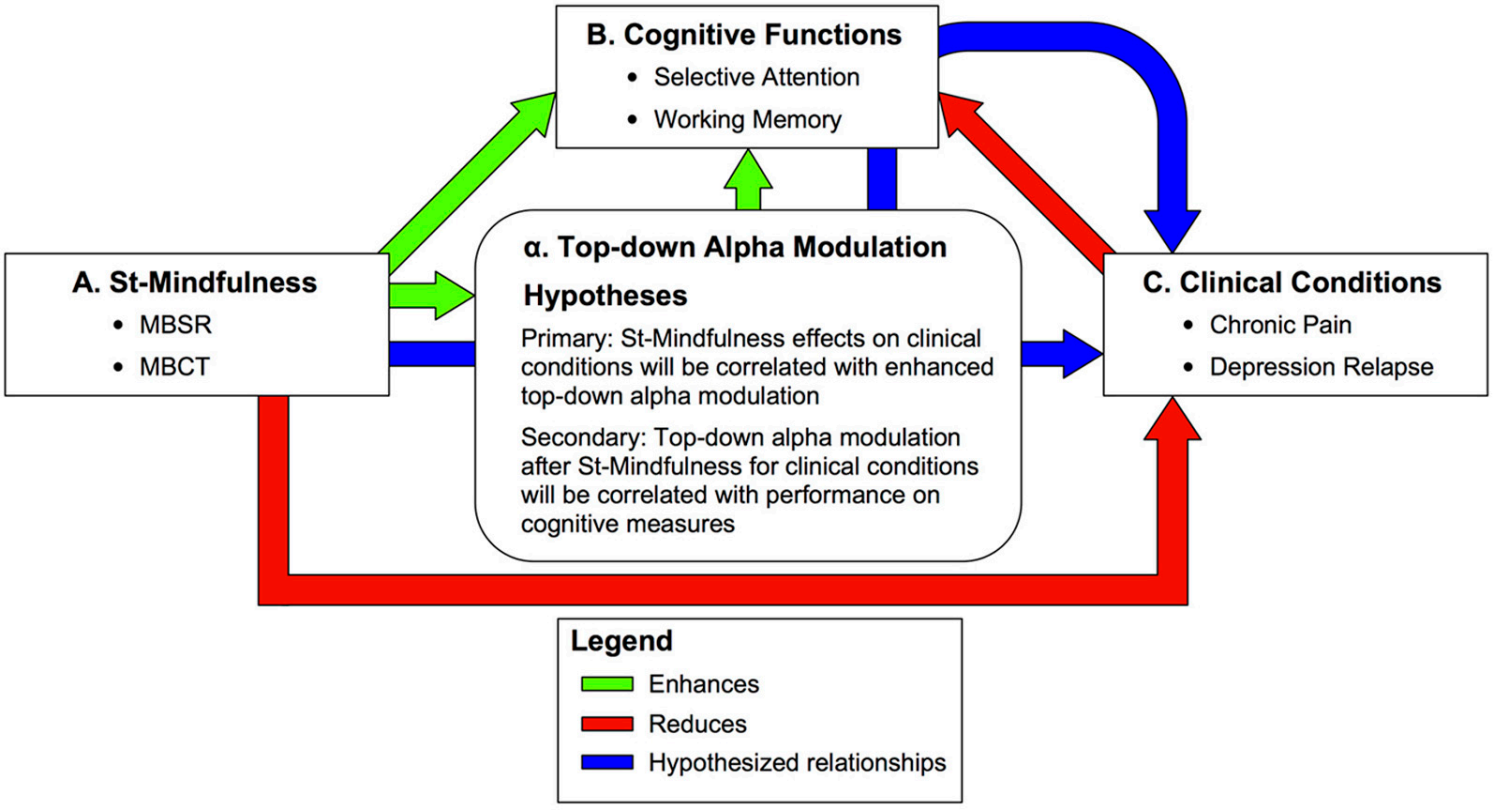
**Summary of predictions on the effects of Standardized Mindfulness Training (ST-Mindfulness) on cognitive and clinical conditions through top–down alpha modulation. Green arrows**—enhanced functions **A** → **B:** ST-Mindfulness enhances working memory (WM) (e.g., Jha et al., 2010; Van Vugt and Jha, 2011) and cued selective attention (e.g., Jha et al., 2007; for a related task, see also Jensen et al., 2012a,b). α → **B**: Top–down alpha modulation is associated with enhanced WM performance (e.g., Tuladhar et al., 2007; Jensen and Mazaheri, 2010; Van Dijk et al., 2010) and enhanced sensory perception in selective attention tasks (Kelly et al., 2009; Jones et al., 2010; Foxe and Snyder, 2011) with TMS studies suggesting alpha is causally implicated in memory (Sauseng et al., 2009) and perceptual tasks (Romei et al., 2010) **A** → α: ST-Mindfulness enhances attentional modulation of alpha rhythms in SI (Kerr et al., 2011a,b). **Red arrows**—reduced functions **A** → **C**: ST-Mindfulness reduces distress in chronic pain (e.g., Sephton et al., 2007; Gaylord et al., 2011; Schmidt et al., 2011) and reduces risk of depression relapse (e.g., Teasdale et al., 2000a,b; Segal et al., 2010). **C** → **B**: WM and selective attention performance are reduced in chronic pain (e.g., Gijsen et al., 2011; Moore et al., 2012) and depression (e.g., Goeleven et al., 2006; Roiser et al., 2012). **Blue arrows**—hypothesized mechanisms of ST-Mindfulness. *Primary*: **A** → α → **C**: We predict that 8-week ST-Mindfulness training elicits enhanced top–down alpha modulation in sensory cortex that corresponds to improved clinical conditions including chronic pain and depression. *Secondary*: **A** → α → **B** → **C**: We further predict that top–down alpa modulation after ST-Mindfulness for clinical conditions will be correlated with performance on cognitive measures including selective attention and working memory.

In what follows, *Part one* describes how the specific localized body-focused attentional practice seen in ST-Mindfulness led us to test the hypothesis that ST-Mindfulness enhances attentional control over a localized alpha rhythm in primary somatosensory cortex (SI). *Part two* outlines the basis for generalizing our hypothesis to predict that ST-Mindfulness enhances the ability to modulate alpha rhythms across sensory neocortex in an internally directed, top-down manner for forms of regulation such as selective attention and working memory. *Part three* considers the evidence related to our hypothesis that ST-Mindfulness's positive effects on distress and mood in trials of chronic pain and depression relapse are correlated with its efficacy in enhancing top-down modulation of alpha rhythms in sensory neocortex in sensory-attention and working-memory paradigms. (See Figure 1 for a summary of the framework). *Part four* reviews our computational neural modeling results that provide a cellular and network interpretation of possible neural mechanisms generating alpha in sensory cortex and the implications of this interpretation for understanding alpha modulation during ST-Mindfulness training. *Part five* considers the implications of this framework for scientific understanding of mindfulness meditation. Description of our parallel hypothesis, that this training also serves as a first step in learning to control thalamocortical alpha oscillations in non-sensory neocortex loops, is beyond the scope of the current work and will be considered in depth elsewhere.

## Part 1: initial evidence in the somatosensory system that ST-mindfulness enhances top-down alpha modulation

### ST-mindfulness training of localized attention to body awareness

In the first 2 weeks of ST-Mindfulness practice, body-focused attention is highly localized: subjects carry out a forty-minute daily attentional scan of 32 different parts of the body (referred to as the “bodyscan”), directing a relaxed attentive focus toward each part, beginning with the toes and concluding with the top of the head (Kabat-Zinn, 1990; Segal et al., 2002). Subjects are asked to attend to somatic sensations at a high level of detail, as seen in the instructions to subjects at the beginning of their first sustained meditative practice in which they are asked to focus on “the big toe (in the left foot) and, if you can, the little toe, not moving them, but just feeling them individually and perhaps the toes in between (Kabat-Zinn, 2005).” The localized attention to sensations in a specific body area is continued in the sitting meditation taught in the last 4 weeks of ST-Mindfulness. This focus can be seen in the MBCT guide for patients dealing with depression (Williams et al., 2007), written by the clinical scientists who developed the approach (Teasdale et al., 2000a,b; Ma and Teasdale, 2004; Segal et al., 2010), in which the practice of sitting in mindfulness meditation is introduced as a practice of focusing somatic attention on the location in the body where the practitioner finds the sensations of the breath to be “most *vivid* and *distinct*.” The focus on localized somatosensory attention is also trained in the (more briefly practiced) mindful yoga and walking meditation, in which students learn to focus mindful attention on sensations in the feet (Segal et al., 2002). This emphasis on localized somatic attention is also described by subjects in qualitative studies (Mason and Hargreaves, 2001; Morone et al., 2008a,b; Kerr et al., 2011a,b; Langdon et al., 2011). Given this emphasis on locally focused somatic attention, still unanswered is the question of why ST-Mindfulness is taught in this manner? How does this specific practice lead to positive clinical outcomes in chronic pain and depression relapse?

### Evidence of attentional modulation of the 7−14HZ alpha rhythm in SI in healthy normal subjects

Higher-order cognitive processes including selective attention and working memory are enabled by the basic ability to filter irrelevant sensory information while focusing on relevant information (James, 1890; Foxe and Snyder, 2011) Without this ability to screen irrelevant inputs, the flood of sensations would diminish our ability to carry out basic cognitive operations.

Recent discoveries point to spontaneous alpha oscillations (7–14 Hz) as playing a mechanistic role in filtering sensory inputs: Anticipatory increases in the alpha rhythm in primary sensory cortex before the arrival of a stimulus are hypothesized to inhibit or “gate” processing of non-attended stimuli (Foxe and Snyder, 2011), while alpha is held constant in the specific location in the contralateral primary sensory map corresponding to the attended location and is thus specifically spared from the inhibitory impact of broad alpha increases. There are several synaptic and cellular level properties engaged by alpha oscillations that could mediate their proposed suppression of local sensory throughput (see, for example, Chung et al., 2002; Osipova et al., 2008; Jones et al., 2009; Jensen and Mazaheri, 2010). There is, however, no consensus on how this modulation is achieved (in section “Part-4: Predictions from our Computational Neural Model on Neural Mechanisms Underlying Enhanced Alpha Modulation in ST-Mindfulness,” we describe a computational model designed to shed light on physiological mechanisms underlying alpha modulation).

Initial support for body-focused attention as a possible mechanism underlying ST-Mindfulness comes from our experiment (Jones et al., 2010) showing that in normal healthy subjects, locally focused somatic attention exerts specific changes in localized alpha rhythms in the primary somatosensory map: when the subject is cued to attend to the hand, alpha power is *decreased* in the contralateral hand map in SI. Alpha power is *increased* in the contralateral hand map when the subject is cued to attend to a different body location. In the somatosensory domain, studies by other groups replicating and extending our finding have discovered a general functional role for the somatosensory alpha rhythm as a filtering mechanism distracting or inputs in a broad range of information processing tasks [including selective spatial attention (Haegens et al., 2011; Van Ede et al., 2011) and working memory (Spitzer and Blankenburg, 2011)].

### Evidence that ST-mindfulness enhances attentional modulation of alpha in SI

**Figure 2:**
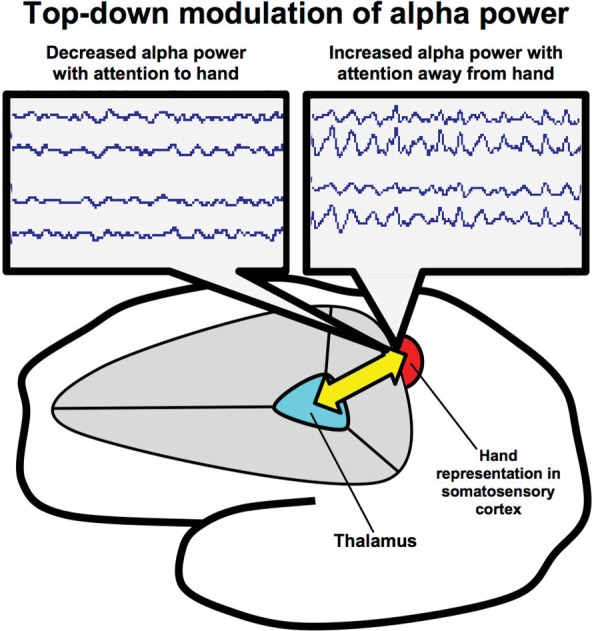
**Thalamocortical circuitry involved in ST-Mindfulness and somatosensory attentional modulation of alpha rhythms.** Attentionally driven increases in alpha rhythm power broadly suppress sensory throughput in the *unattended area* (via thalamocortical mechanisms); spatially specific suppression of alpha facilitates sensory throughput in the *attended area* from the sensory periphery to the thalamus and on to the cortex.

Following the discovery that alpha rhythm modulation is correlated with sensory filtering during body-sensation focused attention, we probed whether subjects trained in ST-Mindfulness would show enhanced top-down modulation of a localized alpha rhythm in SI. We were especially interested in measuring alpha rhythm responses to different visual cues in primary SI (see Figure 2). Given their training in localized attention to body sensations, would subjects trained in ST-Mindfulness show enhanced top-down anticipatory control over the somatotopic alpha rhythm, after a visual cue (to attend “foot” or attend “hand”) prior to a stimulus?

**Figure 3:**
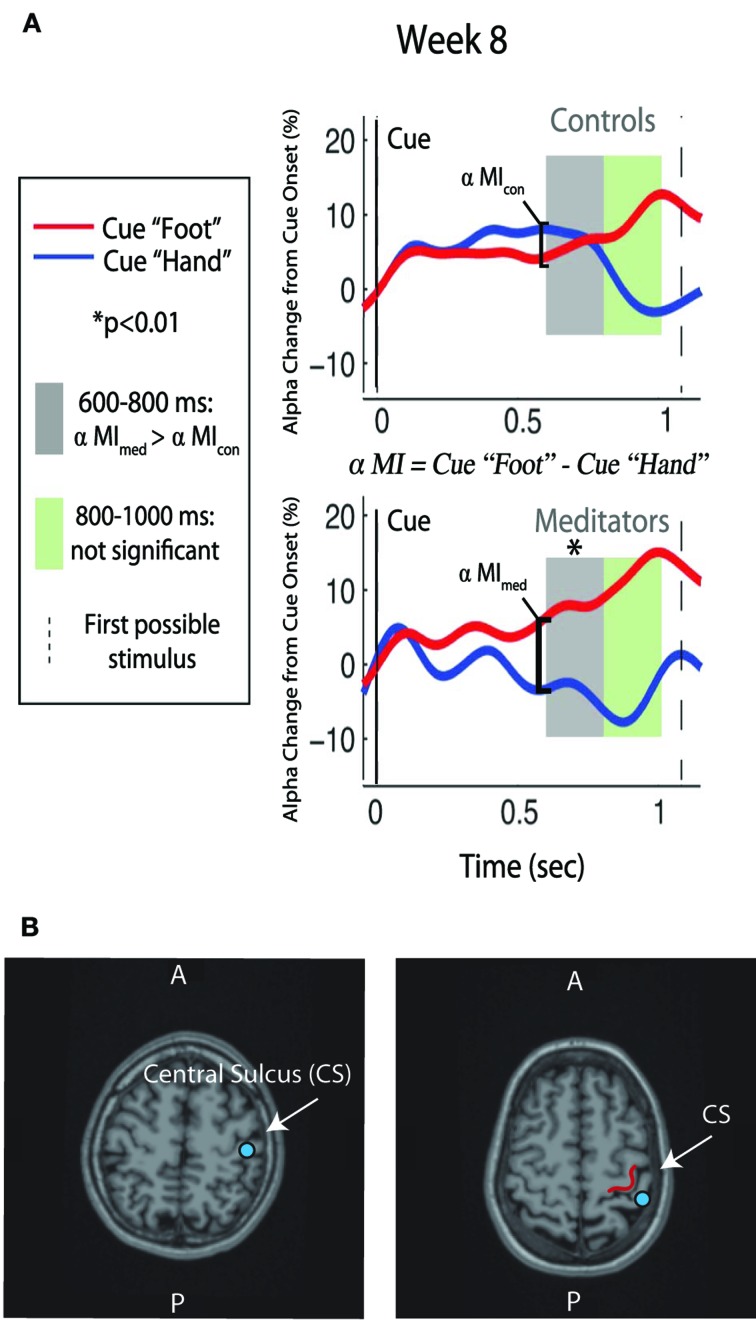
**Alpha modulation and ST-Mindfulness training. (A)** Compared to non-meditators, ST-Mindfulness subjects' exhibit greater alpha differentiation between attend-hand vs. attend-foot conditions in the early post-cue period [600–800 ms, indicated by shaded region; originally published in Kerr et al. (2011a,b). Permission to use figure received from *Brain Research Bulletin*]. **(B)** From two participants, illustration of SI localization, with equivalent current dipole (blue dots) overlaid on MRI brain structure images proximal to the omega shape in the anterior bank of the post-central gyrus.

We hypothesized that after 8 weeks of training, ST-Mindfulness subjects would show enhanced attentional regulation of the somatosensory alpha rhythm by achieving a faster and larger dissociation between alpha measured in the SI hand map after the cue to attend toward versus away from the hand. To test our hypothesis, healthy participants were randomly assigned to 8 weeks of ST-Mindfulness (MBSR) or to a wait-list control. Using magnetoencephalography (MEG) to localize alpha in SI, we found that the ST-Mindfulness group showed significant gains in the ability to regulate alpha (Kerr et al., 2011a,b): the mindfulness group, which had just completed 8 weeks of localized somatic attention training, used attention to achieve faster and greater control over a localized measure of alpha power in the contralateral SI handmap. That is, the ST-Mindfulness group's neuronal response to a cue to attend toward or away from the left index finger and was significantly faster and greater than that of the control group (see Figure 3) with ST-Mindfulness practitioners performing better in resetting their sensory filters in anticipation of a touch stimulus, as a response to changing contextual cues.

Importantly, this finding is in line with reports of ST-Mindfulness and related practices enhancing somatosensory-attention and perceptual processes (Kerr et al., 2008; Fox et al., 2012; Mirams et al., 2012).

## Part 2: the generalizability of ST-mindfulness as an enhancer of top-down alpha modulation in other sensory systems

### ST-mindfulness enhances attentional modulation of alpha in other sensory areas

Alpha rhythms in other sensory systems in the cortex (e.g., visual and auditory systems) follow the same general principles as those described above for the somatosensory system with TMS studies causally linking experimentally induced changes in alpha in changes in perception (Romei et al., 2008, 2010), suggesting that our ST-Mindfulness theoretical framework should be generalizable to include top-down modulation of alpha rhythms across sensory neocortex (see Figure 1 for a summary of the framework) (Worden et al., 2000; Thut et al., 2006; Rihs et al., 2007; Kelly et al., 2009; Banerjee et al., 2011).

Results from a recent study in the visual domain support a broader role for top-down alpha modulation in ST-Mindfulness. Specifically, (Jha et al., 2007) found that meditators trained in a variant of ST-Mindfulness showed improved reaction time in a cued visual spatial selective attention paradigm similar to the one tested in (Kerr et al., 2011a,b). These results suggest that somatic attentional modulation in ST-Mindfulness may bootstrap a more generalized improvement in selective spatial attention in visual and auditory modalities. These results are supported by several tests correlating ST-Mindfulness with enhanced attentional performance and reduced errors in tests of visual selective attention (Semple, 2010; Jensen et al., 2012a,b), although not all studies are positive (see for example Anderson et al., 2007).

### Evidence that ST-mindfulness-related improved performance in cognitive tasks is due to enhanced top-down modulation of alpha

Other forms of top-down alpha modulation are also relevant for understanding mechanisms underlying ST-Mindfulness. Working memory, for example, is an internally focused cognitive task that is reported to improve with ST-Mindfulness training (Jha et al., 2010) (see also Van Vugt and Jha, 2011). Working memory is also highly correlated with top-down alpha modulation (Jensen and Mazaheri, 2010). Multiple studies have shown that the ability to broadly increase alpha power over sensory regions during a memory retention period is significantly correlated with the subsequent performance on a working memory task (Tuladhar et al., 2007; Meltzer et al., 2008; Van Dijk et al., 2010). As memory load increases, so does alpha power over sensory processing areas [this result has also been obtained in short-term memory tasks including (Jensen et al., 2002)]. These reports suggest that increased alpha power facilitates working (and short-term) memory processes by taking irrelevant sensory processing regions offline, with at least one TMS study suggesting induced changes in alpha rhythm over parietal-occipital cortex are causally implicated in predicted alterations in memory performance (Sauseng et al., 2009).

Our theory predicts that ST-Mindfulness's localized focus on somatic sensations, facilitates generalized enhancement in top-down control over sensory alpha which gives ST-Mindfulness subjects an enhanced ability to regulate cognitive performance over parameters such as working memory. The basis for this hypothesis comes from studies showing that the same sensory alpha power that is used to inhibit irrelevant sensory-information can also be used to facilitate better control over *internally* focused attention (Chun et al., 2011; Waldhauser et al., 2012). This effect is most apparent in studies correlating alpha modulation with internally focused memory selection (e.g., Bauml et al., 2008; Waldhauser et al., 2012).

Based on these prior studies, our framework predicts that ST-Mindfulness practitioners will show an enhanced ability to use sensory alpha modulation to facilitate behaviorally relevant internal stimuli (e.g., active working memory processes) by increasing alpha to block competing sensory processes (Waldhauser et al., 2012). The framework also predicts that ST-Mindfulness practitioners' are able to decrease rumination by using sensory alpha to *suppress distracting, irrelevant internal stimuli* (e.g., ongoing negative ruminative memories or associations) by attending to a sensory stimulus such as the breath. The resulting focal sensory alpha decreases the salience of the internally focused ruminative attention [see also Chun et al.'s (2011) account of internal focused cognitive processes].

## Part 3: clinical implications of ST-mindfulness's role as an enhancer of top-down alpha modulation in chronic pain and depression relapse

ST-Mindfulness's most prominent clinical benefits can be seen in trials showing it significantly reduces the risk of depression relapse (with high risk patients showing the greatest benefit) and it reduces pain-related distress and increases mood and quality of life in difficult chronic pain conditions such as fibromyalgia. Here, we provide a brief discussion of how the somatosensory attentional training mechanism described above is thought to play an important role in mindfulness' effects on depression and chronic pain (see also Figure 1).

### The relationship between chronic pain, ST-mindfulness, and attention

In chronic pain situations, nearly all studies of ST-Mindfulness show relief of pain-related distress and increased mood. Some studies show direct relief of pain (see Morone et al., 2008a,b for example), although this finding seems to be more apparent in experimental pain paradigms with normal healthy subjects (Zeidan et al., 2010) than in chronic pain patients. These results pose a puzzle because the type of spatial-attentional modulation engaged in by ST-Mindfulness subjects does not appear to engage or modulate the *affective component* of the pain experience or related brain regions, such as the anterior insula and the amygdala (Kulkarni, 2005). Yet, the end result of this somatic attentional practice is a positive change in pain-related affect.

In order to understand how the body-sensation focused attentional practice learned in ST-Mindfulness decreases distress, it is important to understand how somatosensory attention is disregulated or “biased” in chronic pain. Chronic pain patients demonstrate attentional bias that affects their ability to process body related sensations according to their relevance and also affects their general ability to selectively attend to specific stimuli or to carry out complex cognitive tasks that require control over attentional deployment (Gijsen et al., 2011; Moore et al., 2012). This attentional bias leads patients to attend excessively to the painful area (Moseley et al., 2005), resulting in both hypersensitivity in the painful area and hypoesthesia with deficits in tactile perceptual processing (Moriwaki and Yuge, 1999) in other areas. Our framework makes sense of these findings by relating both hypoesthesia and hyperesthesia to decreased ability to modulate alpha. We hypothesize that these related areas of painful hypersensitivity and tactile hypoesthesia are, in part, maintained by the continued, locked engagement of attentional alpha biasing in a somatosensory cortical area. In effect, this anticipatory alpha biasing system has become permanently oriented toward the painful areas.

While normal subjects are able to shift attention away from pain during a visual attention task, chronic pain patients cannot carry out attentional filtering of the competing pain stimulus. That is, normal subjects use alpha modulation to filter out pain sensations (Babiloni et al., 2003, 2006) and can reduce brain activity in pain-related regions including SI by redirecting attention during a painful stimulus to a competing cognitive task (Seminowicz and Davis, 2007; May et al., 2012). Chronic pain patients, however, are unable to carry out this attentional modulation of pain: unlike normal subjects, whose pain decreases when they are carrying out a demanding attentional task in a competing sensory modality, pain patients do not demonstrate top-down modulation of pain intensity (Snijders et al., 2010). Based on these results, we hypothesize that this lack of attentional flexibility in modulating pain is reflected in chronic pain patients' decreased ability to carry out top-down modulation of alpha rhythms in response to moment-by-moment changes in context. We predict that ST-Mindfulness training in attention to localized somatic sensations enhances the ability of chronic pain patients to carry out top-down modulation of sensory cortical alpha in response to moment-by-moment changes in task-related attentional demands.

According to our theoretical framework, the somatosensory attentional training in ST-Mindfulness may work in chronic pain by “unsticking” the chronically stuck sensory attentional system. For example, as a method of facilitating this “unsticking,” it may be that the ST-Mindfulness body scan practice teaches subjects to first engage (by directing attention *toward*) and then disengage (by withdrawing attention *from*) every body area. By this process of repeatedly engaging and disengaging alpha dynamics across the body map, according to our alpha theory, subjects are relearning the process of directly modulating localized alpha rhythms. In many pain patients, this attentional process allows patients to directly attend to the painful area. According to our hypothesis, it is this direct attentional training toward the pain that “de-biases” the system and frees up attentional resources that were previously stuck in patterns used to cope with the ongoing pain sensations. We hypothesize that ST-Mindfulness subjects would show increased ability to modulate alpha in an anticipatory tactile attention paradigm similar to that used in (Kerr et al., 2011a,b). Attentional resources previously dedicated to maintaining pain-related biases prior to ST-Mindfulness become available to filter distractions, enhance signal-to-noise ratio and disengage from irrelevant sensory inputs in a manner that reduces distress and improves mood and quality-of-life. Given this alpha modulation mechanism, we would not expect ST-Mindfulness training to completely eliminate the pain experience in chronic patients, as it would likely not address a baseline level of pain driven by underlying pain mechanisms such as central sensitization that are present in the absence of competing sensory attentional tasks. Rather, we would expect chronic pain patients receiving ST-Mindfulness training to report enhanced ability to attend to moment-by-moment attentional task demands in their daily life as reflected in increased self-reported quality of life and mood (Grossman, 2004; Sephton et al., 2007).

### The relationship between prevention of depression relapse, ST-mindfulness, and attention

Patients with depression and remitted depression show information processing deficits in perception, attention, and memory (Roiser et al., 2012). In particular, they show deficits in filtering distracting stimuli (Pasto and Burack, 2002), disengaging from irrelevant stimuli (Dietl et al., 2001) and learning to discriminate signal from noise (Kemp et al., 2009).

The significance of these deficits for emotional function can be seen most clearly in studies of depressed patients' and formerly depressed patients' moment-by-moment processing of facial emotional expression. Depressed patients and formerly depressed patients show perceptual and attentional bias for sad faces and bias against positive faces (Goeleven et al., 2006; Roiser et al., 2012). The significance of this bias is that the ability to read emotions during social encounters is impaired. A deficit in decoding facial expression in depressed and formerly depressed patients is thought to have adverse consequences for interpersonal interactions such as the ability to perceive and actively experience social support (Bistricky et al., 2011).

Importantly, basic sensory filtering is relevant to decoding facial emotional expressions. Alpha gating processes similar to those described above are reported in facial emotion tasks (Chen et al., 2010). Alpha increases are used to control the flow of information in the brain by gating stimuli to task-irrelevant sensory areas. Based on these studies, we hypothesize that ST-Mindfulness in subjects at high risk of depression relapse would bring about improved sensory alpha modulation in a facial emotion perception paradigm (Chen et al., 2010) and in a tactile working memory paradigm similar to (Spitzer et al., 2010). A positive result would validate our broader theory that in people at high risk of depression relapse, attentional engagement with localized somatic sensations may be useful for retraining basic sensory filtering processes required to support perception of emotional facial expressions.

The localized somatosensory attentional focus of ST-Mindfulness may also be important for helping to gate negative internally focused cognitions such as rumination or catastrophizing, since there is an ongoing competitive process between internally focused cognitive/memory tasks and sensory attentional tasks (Chun et al., 2011). As such, our framework predicts that learning to focus sensory attention on the breath and on body sensations should help decrease the salience of internally focused ruminative thought-streams. A more localized somatic attentional focus, according to our framework, will be correlated with higher efficacy in achieving decreases in sensory cortical alpha that are in turn causally related to decreases in internally focused rumination. In chronic pain, we similarly hypothesize that a sensory attentional focus may enable pain patients to “gate” catastrophizing cognitions (by which some pain patients attach special meaning to their pain, endorsing items such as, “I cannot stop thinking about how much it hurts”). More generally, by training in voluntary attentional modulation of sensory processes, ST-Mindfulness may restore attentional freedom to persons with chronic pain or depression that have been trapped in internally focused negative cognitions.

## Part 4: predictions from our computational neural model on neural mechanisms underlying enhanced alpha modulation in ST-mindfulness

According to the theory presented here, body-sensation focused attentional practice facilitates enhanced top-down alpha modulation in ST-Mindfulness in a manner that may be helpful for chronic pain and for preventing depression relapse. We propose this enhanced modulation depends in part on the ability to dynamically, flexibly alternate between alpha increases in broad sensory areas corresponding to unattended stimuli and localized attention-driven suppression of alpha increases in the sensory cortical map corresponding to the attended location. The ability to carry out top-down modulation of alpha on both a localized scale and across entire sensory cortical areas requires a dynamic, responsive underlying neuronal control mechanism.

We have developed a biophysically principled computational neural model in SI that gives insight into the cellular and network level mechanisms inducing alpha and can help us visualize how ST-Mindfulness training may enhance the ability to flexibly carry out these localized and broad modulatory alpha effects.

### Model alpha rhythms are produced by the interaction of two 10HZ thalamocortical inputs from specific and non-specific thalamic nuclei

Our model of a cortical column in primary somatosensory neocortex contains excitatory pyramidal neurons and inhibitory interneurons across cortical layers. In this model, the 10 Hz alpha rhythm is characterized as part of a two-component SI rhythm called “mu” in humans that also contains a (15–29 Hz) beta component (Jones et al., 2009).

**Figure 4:**
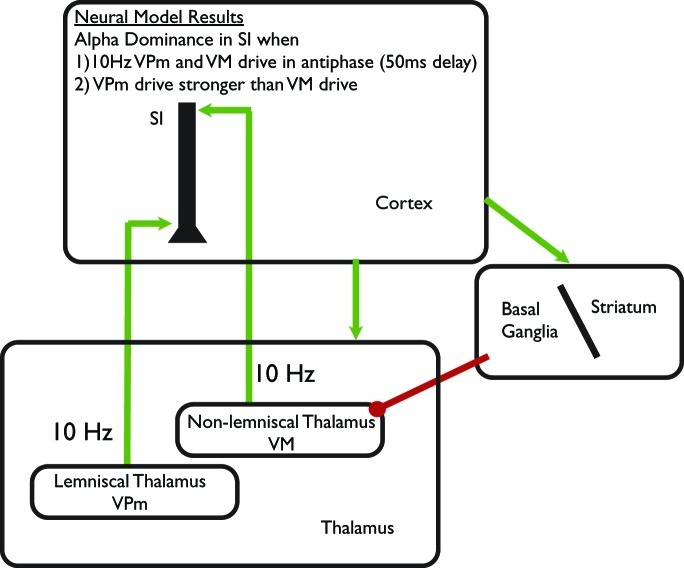
**Schematic illustration of computational neural modeling predictions on the origin of alpha.** Green arrows represent excitatory synaptic connections and red circles inhibitory synaptic connections. We hypothesize that focal changes in alpha can be achieved by modulation of the lemniscal thalamic Ventral-Posterial medial (VPm_) pathway to SI, while diffuse regulation can be achieved through modulation of non-specific Ventral-Medial (VM) thalamic drive. The VM thalamic nucleus is under direct inhibitory control of the Basal Ganglia/Striatum circuit, which is influenced by the prefrontal cortex. These pathways suggests alpha modulation occurs through alteration of prefrontal-basal ganglia—thalamocortical circuits in ST-Mindfulness practitioners (see discussion in “Part-4: Predictions from our Computational Neural Model on Neural Mechanisms Underlying Enhanced Alpha Modulation in ST-Mindfulness,”).

The model was based on accurately simulating brain signals measured non-invasively in humans with MEG and the results have been shown to be tightly correlated with experimental MEG data in multiple studies (Jones et al., 2007, 2009; Ziegler et al., 2010). The model results led to the specific prediction that cortical alpha rhythms are generated by two distinct 10 Hz thalamocortical drives to cortex that terminate in different cortical layers. These exogenous excitatory synaptic drives are representative of *lemniscal thalamocortical* input to granular/infragranular layers and *non-specific thalamic input* to supragranular layers (see schematic illustration in Figure 4). The drives produce post-synaptic current flow within the large spatially extended and aligned pyramidal neurons in the cortex to reproduce the MEG measured rhythm (Jones et al., 2009). Model results show that the emergence of an alpha (or beta) rhythm at a specific point in time depends on two key parameters: the delay between the two drives on each cycle of the rhythmic (100 ms period/10 Hz) drive, and the relative efficacy of the granular/infragranular vs. supragranular drives. Alpha oscillations are dominantly expressed when (1) the delay between the rhythmic drives is asynchronous near anti-phase [i.e., 50 ms, in agreement with laminar recordings (Bollimunta et al., 2011)] or (2) the efficacy of the granular/infragranular drive is greater that the supragranular drive (see Figure 8 in Jones et al., 2009). Our model-based hypotheses extend prior theories on the origin of cortical alpha rhythms in awake humans that are generally assumed to depend on 10 Hz thalamic drive, and cortical thalamic interactions (Da Silva et al., 1973; Hughes et al., 2004; Hughes and Crunelli, 2005).

### Hypothesis: ST-mindfulness creates precision in the relative timing and efficacy of the specific and non-specific thalamocortical drive

Based on this computational model, we hypothesize that ST-Mindfulness creates increased precision in the timing and efficacy of drives from lemniscal and non-lemniscal thalamic nuclei. Interactions between specific thalamic nuclei (VPm), and non-specific thalamic nuclei are particularly attractive as control centers to simultaneously decrease alpha locally and increase alpha more broadly. The fine topographically specific arrangement of thalamocortical connections from VPm is well suited to adjust alpha rhythmicity locally, while the more diffuse connections from non-lemniscal sources to supragranular layers and SI and other cortical areas is ideal for broader modulations such that refined control of thalamocortical drive enables finer top-down attentional control and filtering of both spatially localized sensory information and whole sensory areas in neocortex (Jones, 2001). A candidate area for the non-lemniscal thalamic nucleus that projects to SI is the Ventral Medial (VM) thalamus, as depicted in Figure 4, which has been shown to project nearly exclusively to the supragranlular layers in SI (Herkenham, 1980; Desbois and Villanueva, 2001; Rubio-Garrido et al., 2009; Sherman and Guillery, 2009; Theyel et al., 2010).

Enhanced top-down alpha modulation by ST-Mindfulness in other sensory modalities could also be achieved through thalamic regulation as the close vicinity of thalamic nuclei to one another suggests the possibility that their relative timing and efficacy could be rapidly adjusted in relation to one another (Theyel et al., 2010). The precise mechanisms of such thalamic modulation are beyond the current predictions of the model but likely involve basal forebrain cholinergic system activation and cortical feedback from prefrontal cortex or from striatal/basal ganglia influences to the distinct thalamic nuclei engaged during attentional modulation (see Figure 4). The involvement of the prefrontal cortex is in line with prior studies of ST-Mindfulness showing changes in alpha asymmetry in prefrontal areas (Davidson et al., 2003) as well as differences between dorsal prefrontal activations in ST-Mindfulness groups (Farb et al., 2007).

The view of ST-Mindfulness as enhancing cortical alpha modulation via thalamic mechanisms extends earlier theories of thalamic dysrhythmia in resting alpha rhythms as a pathological mechanism in chronic pain and depression (Llinas et al., 1999). It is also related to earlier reports showing generalized increases in resting alpha in advanced meditators (Kasamatsu and Hirai, 1966; Cahn and Polich, 2006). However, unlike these earlier theories, our framework hypothesizes ST-Mindfulness enhances the ability to carry out real-time modulation of thalamocortical timing in response to changes in behavioral context, rather than tonic levels of ongoing alpha rhythms.

### Hypothesized ST-mindfulness regulation of non-specific thalamic nucleus VM is supported by its putative role in circuits involving chronic pain and depression

Applying the model to the sensory attentional biases described above in chronic pain and depression relapse, we would hypothesize that in chronic pain, pervasive abnormalities in somatosensory attention would be reflected in disordered and inflexible modulation of thalamic drive putatively connecting specific and nonspecific cells in thalamus to SI, indicating a decreased ability to use attention to modulate these drives. A possible disregulation of the VM thalamic nucleus, the non-specific nucleus we hypothesize may be specifically involved in alpha modulation in SI, is directly supported by experimental evidence. Most importantly, the VM nucleus is known to be involved in diffuse, non-lemniscal, non-topographically specific pain processes (Desbois and Villanueva, 2001; Monconduit and Villanueva, 2005).

In subjects at high risk of depression relapse (Desbois and Villanueva, 2001), we would also expect pervasive abnormalities in thalamic coordination across sensory cortical regions. Abnormalities in VM regulation would also be directly connected to circuits involved in depression. In particular, depression involves disruption in the dopaminergic system in the striatal/basal ganglia network, which provides an inhibitory projection directly to VM and other thalamic nuclei (Di Chiara et al., 1979; Deniau et al., 1992). Thus, more efficient gating of the VM-SI pathway with ST-Mindfulness would fit with the model predictions on mechanisms of alpha modulation and provide insight as to how it elicits beneficial changes in chronic pain and depression.

The model predicts that ST-Mindfulness gains in localized and broad sensory modulation are achieved by enhancing precision in thalamocortical timing via increased control over both localized spatial attention (used in the ST-Mindfulness body scan) derived from lemniscal thalamic VPm and broader scale attentional modulation of an entire sensory modality via non-specific, non-lemniscal nucleus, possibly VM (e.g., as when, in ST-Mindfulness, practitioners learn to view distressing thoughts as internally generated events that arise and fall in a manner analogous to sensory stimuli).

In ST-Mindfulness, this dual modulation of both highly topographically specific and broad sensory processes can be seen in the sequence of practice described by Williams (Williams et al., 2006), in which practitioners first learn a detailed body scan practice of “moving a focused spotlight of attention from one part of the body to another”; from this practice, practitioners learn how *body sensations change and fluctuate* from moment to moment and they learn how to observe the arising and passing of challenging *body sensations*. Thus, our biophysical model is in line with the idea that ST-Mindfulness, with its cultivation first of a narrow, somatotopically focused attention that ultimately enables broader modulation of the sensory field which in turn enabled a more sustained yet homeostatically regulated attention (i.e., that does not cause emotional flooding) to distressing thoughts, feelings, and sensations.

## Part 5: significance of this framework for the science of mindfulness meditation

While many researchers have regarded ST-Mindfulness as a form of cognitive training (Hamilton et al., 2006; Hollon and Ponniah, 2010), the alpha modulation framework described here can help us reorient our understanding of ST-Mindfulness as a *sensory-attention-cognitive* practice. In this view, it is useful to think of ST-Mindfulness as enhancing top down alpha modulation of gain control. No longer viewed as a simple noise reduction technique, top-down regulation of gain control is now thought to play an important role in regulating emotion (Lachat et al., 2012) and cognition (Haegens et al., 2010).

In the next sections we describe several specific ways that a focus on top-down alpha modulation as a regulator of gain control helps to frame key findings in the mindfulness literature related to regulating cognition and emotion.

### Initial training in awareness of mental processes: the role of somatic feedback in somatically focused mindfulness

According to the framework presented here, top-down alpha modulation of gain control plays a key role in guiding ST-Mindfulness practitioners to recognize and modulate their own attentional spotlight, especially in somatically focused meditation practice. During the body-scan and breath-focused awareness, ST-Mindfulness and other mindfulness-trained subjects frequently report perceptual feedback from the fingers, toes, abdomen, etc. (see, for example, Kerr et al., 2011a,b; Fox et al., 2012; Mirams et al., 2012). Data from our alpha modulation study suggest that these perceptions occur when the practitioner's sensory attention “spotlights” input from a specific somatic area, with the spotlight being maintained by enhanced alpha gating of unattended stimuli. These spontaneous stimuli provide a perceptual correlate for practitioners to detect where the mind is focused. This detection may allow for the regulation of mind-wandering, specifically, when the mind wanders from its somatic attentional focus during meditation.

We predict that this direct experience in detecting somatic mind-wandering gives practitioners facility in perceiving the mind's attentional focus when it is directed to other sensory modalities, and, importantly, when it is directed toward internally occurring thoughts. This view is in line with what is explicitly taught in mindfulness training: to regard thoughts as “mental events” that arise and pass in the mind in a manner analogous to spontaneously occurring body sensations. Thus, this skill in detecting the focus of mental attention may be an integral part of the broader training sequence [that includes one's own perceptions, emotions, and thoughts; for review of this proposed metacognitive transformation (Bishop, 2004; Shapiro et al., 2006)].

### Emotion perception and emotion regulation

The notion that ST-Mindfulness enhances alpha rhythm modulation of gain control is complementary with the behavioral process of interoception. Interoception is defined as the perception of internal visceral sensations such as heartbeat, gastric sensations and sensations of breathing that are often laden with emotion. Numerous reviews (Corcoran et al., 2009; Holzel et al., 2011) have identified interoception as an important mechanism that facilitates cognitive and emotional regulation in ST-Mindfulness. ST-Mindfulness is thought to work, in part, by enhancing attentional access to emotionally driven visceral sensations encoded in the insular cortex. Enhanced interoception in ST-Mindfulness is thought to facilitate better understanding and processing of emotional reactions to external stimuli and events.

ST-mindfulness' emphasis on directly regulating gain control in practices such as the body scan may give practitioners an important skill for regulating visceral interoception. That is, alpha modulation of gain control may be an important resource as practitioners learn how to engage emotion-laden sensations in the chest, throat and stomach without being flooded by emotion. In the body scan, participants first receive instruction in modulating gain control as they learn to focus on, and then, crucially, to disengage from both “cold” and emotionally “hot” sensations. By learning to shift the attentional spotlight with equanimity across both challenging and non-challenging somatic areas, practitioners learn to “de-bias” their attention to emotion-laden sensations. Their enhanced ability to use alpha modulate the “volume” of a specific sensory input thus may allow practitioners to focus on sensations laden with emotional significance with limited reactivity. In this sense, practitioners learn to treat these emotion-laden sensations in a similar manner to sensations that do not have great emotional significance. This initial regulatory learning provides an important foundation for practitioners' ability to work with and be present to difficult emotional experiences.

### Cultivation of broad attentional focus in mindfulness and the development of flexible emotional and cognitive regulation

Our alpha-modulation hypothesis proposes that initial training in somatosensory alpha power modulation (where there is perspicuous perceptual feedback) becomes generalized with more training across sensory neocortex. An important test of this hypothesis would be to assess the abilities of practitioners of different experience levels in modulating alpha-rhythm activity. We predict that advanced practitioners will exhibit broad and temporally precise alpha modulation.

A potential limitation of this advanced practitioner hypothesis is that such practitioners (many of them with tens of thousands of hours of practice) have been found to engage in meditation techniques that use a more open-ended attentional focus from those learned in the ST-Mindfulness 8-week sequence. That is, while beginner's practices tend to use a localized mindful focused attention (M-FA), advanced practitioners transition toward a *mindful open monitoring* (M-OM) (Lutz et al., 2008) practice that cultivates the ability to disengage from an object that has seized attention, using a broad awareness of the contents of mind without deliberate selection of a primary attentional focus.

Evidence for attentional flexibility in advanced practitioners comes from a recent study showing that advanced meditators were able to disengage from previous stimuli (in an attentional blink paradigm) more quickly after 3 months of intensive, residential M-OM practice, (Slagter et al., 2007). This suggests that the attention of advanced practitioners was no longer captured by a pre-selected target but was able to move to the next task requirement.

As performance on attentional blink tasks appears to depend on the ability to achieve better control over alpha rhythm phase dynamics (Mathewson et al., 2011), it is likely that this form of enhanced top-down alpha was used by M-OM practitioners to improve performance in the attentional blink. More generally, recent reports suggest alpha rhythms are mechanistically involved in the process of permitting the bottom-up switch toward previously unattended stimuli (see Jensen et al., 2012a,b). These reports suggest that alpha modulation does not merely suppress unattended stimuli, but can also enhance bottom-up processing of unexpected events in its role as a filter of information from the thalamus to the cortex.

Top-down regulation of alpha rhythms may thus be a key mechanism by which advanced M-OM practitioners learn to disengage attention in order to maintain greater attentional flexibility that can be especially helpful during stress or when there is emotional or cognitive perseveration. Based on these prior studies, we predict that advanced meditators will show subtle modulation of alpha phase dynamics during an attentional blink paradigm. This result would give preliminary support for our alpha-modulation bootstrap hypothesis, supporting the notion that initial training in somatosensory alpha power modulation becomes generalized with more training across sensory neocortex, and may ultimately result in the use of a broadly tuned awareness to carry out subtle regulation of alpha phase dynamics in a domain general manner.

### How does the sequence practiced in ST-mindfulness lead to the psychological abilities described in the mindfulness literature?

Our sensory-attentional cognitive theory of mindfulness practice proposes that a sequence of psychological abilities is achieved in mindfulness, including emotion perception, metacognition, and finally the release of attentional processes. The achievement of this sequence is thought to be at least partially dependent on improved top-down alpha modulation of gain control achieved by a somatically focused mindfulness practice.

This view of a sensory-attentional cognitive practice sequence is in line with the early Buddhist practice Sutra titled *Four Foundations of Mindfulness* (Analayo, 2004). The Sutra specifies “Mindfulness of the Body” and “Mindfulness of the Breath” as beginners' practices that are thought to enable subsequent gains in “Mindfulness of Feelings” and “Mindfulness of Thoughts.” Working through the sequence from mindfulness of the body to mindfulness of thoughts is believed to enable the ability to be both present and non-reactive to one's internal experiences, such as negative cognitions and strong negative emotions.

## Limitations

There are important limitations to the approach described here related to (1) possible undocumented heterogeneity in ST-Mindfulness training programs (2) poor current understanding of the degree of independence among different proposed behavioral and neural mechanisms (3) methodological limitations related to MEG recording resolution in the SI body map.

1. Heterogeneity across ST-Mindfulness modalities especially in MBSR and MBCT may limit the ability to generalize across the two different mindfulness-based training programs. This is because, despite the standardized weekly format, there are important differences between MBSR and MBCT that may make the approach suggested here, of aggregating across the two modalities problematic. Studies of MBCT have drawn from a well-defined patient population of formerly depressed subjects. Studies of MBSR have recruited across a wide variety of populations. In addition, some MBSR programs have introduced new modules (e.g., dyadic exercises, mindful listening) while in the more thoroughly standardized MBCT there is more cognitive training and a more intentional focus on cognitive and emotional processes than in MBSR. Despite these apparent limits to standardization across mindfulness-based training programs, it should be noted that MBSR and MBCT programs are highly uniform across numerous key features (length of training, use of highly similar formats and curricular materials) and both use nationally recognized certification standards.
2. There is still a very poor understanding whether the mechanism proposed here or whether the broad range of proposed ST-Mindfulness mechanisms explored by other research teams are independent factors or whether there is a high degree of shared variance. Some of the proposed mechanisms include: cognitive regulation, tolerance for uncertainty, training in the transience of all phenomena, attitudinal change. Further tests should be performed assessing the relative independent contribution of the mechanism described here (top-down alpha modulation in SI) to ST-Mindfulness efficacy versus emotion regulation and other mechanisms. If the null hypothesis is favored, this would suggest that ST-Mindfulness's benefits can take place without changes in the ability to carry out localized somatosensory alpha regulation; one interpretation of this result might be that localized somatic attention (which takes up a preponderance of practice time) may not be critical for achieving a positive outcome in ST-Mindfulness, in which case, this component might be able to be reduced, thereby making ST-Mindfulness training more efficient and streamlined.
3. Methodological limitations related to cortical magnetoencapholography measures used in (Kerr et al., 2011a,b). Specifically, we attempted but failed to successfully analyze a condition in which practitioners attended to the toe: the anatomy of the foot map location (running parallel to the surface of the cortex) made this impossible for MEG analysis.

## Conclusion

Our theoretical framework addresses a central question in the science of mindfulness. How does standardized mindfulness (ST-Mindfulness) training in sensory attention to the breath and body sensations used in therapies such as MBSR and MBCT elicit positive changes in apparently unrelated cognitive and affective measures (e.g., mood, rumination, working memory, and pain-related distress?) Initial work by our group provides a preliminary answer, with our finding that ST-Mindfulness training was associated with enhanced top-down modulation of 7–14 Hz cortical alpha rhythm in primary SI.

The review is built on our group's finding (Kerr et al., 2011a,b) that ST-Mindfulness subjects use attention to achieve a faster and significantly larger degree of differentiation of alpha power in the SI handmap, depending on the prestimulus cue. The significance of this finding is described in terms of emerging evidence that *top-down modulation of the thalamocortical alpha rhythm facilitates faster and more sensitive filtering of sensory information in the brain*.

Learning to control alpha oscillations in SI through localized body-focused attention may be a key gateway mechanism for learning to use thalamocortical alpha regulation to suppress irrelevant sensory inputs across sensory neocortex in an internally directed, top-down manner, for forms of regulation such as selective attention and working memory. The framework predicts that ST-Mindfulness's positive effects on mood and distress in trials of chronic pain and depression relapse would be correlated with efficacy in enhancing top-down modulation of alpha rhythms in sensory neocortex. Our computational neural modeling results provide a cellular and network interpretation of these neural mechanisms underlying ST-Mindfulness and top-down alpha modulation. This framework has direct implications for how we conceive of mindfulness practice, as it lays out a predicted *sensory-cognitive* sequence of practice-related gains, whereby localized attention to body sensations enables subsequent gains in emotional and cognitive regulation by enhancing sensory information processing in the brain.

### Conflict of interest statement

The authors declare that the research was conducted in the absence of any commercial or financial relationships that could be construed as a potential conflict of interest.
